# Deep Brain Stimulation of the Subthalamic Nucleus Selectively Modulates Emotion Recognition of Facial Stimuli in Parkinson’s Patients

**DOI:** 10.3390/jcm8091335

**Published:** 2019-08-28

**Authors:** Caroline Wagenbreth, Maria Kuehne, Jürgen Voges, Hans-Jochen Heinze, Imke Galazky, Tino Zaehle

**Affiliations:** 1 Departments of Neurology and Stereotactic Neurosurgery, Otto-von-Guericke University Magdeburg, Leipziger Str. 44, 39120 Magdeburg, Germany (C.W.) (M.K.) (J.V.) (H.-J.H.) (I.G.); 2 Department of Behavioral Neurology, Leibniz Institute of Neurobiology, Leipziger Str. 44, 39120 Magdeburg, Germany

**Keywords:** Parkinson’s disease, deep brain stimulation, subthalamic nucleus, emotion processing, affective priming

## Abstract

Background: Diminished emotion recognition is a known symptom in Parkinson (PD) patients and subthalamic nucleus deep brain stimulation (STN-DBS) has been shown to further deteriorate the processing of especially negative emotions. While emotion recognition generally refers to both, implicit and explicit processing, demonstrations of DBS-influences on implicit processing are sparse. In the present study, we assessed the impact of STN-DBS on explicit and implicit processing for emotional stimuli. Methods: Under STN-DBS ON and OFF, fourteen PD patients performed an implicit as well as an explicit emotional processing task. To assess implicit emotional processing, patients were tested with a lexical decision task (LTD) combined with an affective priming paradigm, which provides emotional content through the facial eye region. To assess explicit emotional processing, patients additionally explicitly rated the emotional status of eyes and words used in the implicit task. Results: DBS affected explicit emotional processing more than implicit processing with a more pronounced effect on error rates than on reaction speed. STN-DBS generally worsened implicit and explicit processing for disgust stimulus material but improved explicit processing of fear stimuli. Conclusions: This is the first study demonstrating influences of STN-DBS on explicit and implicit emotion processing in PD patients. While STN stimulation impeded the processing of disgust stimuli, it improved explicit discrimination of fear stimuli.

## 1. Introduction

Deviant emotion production and recognition have been shown in patients with Parkinson’s disease (PD) when compared to healthy controls and especially impaired recognition of emotional facial expressions has been demonstrated [1,2,3,4]. In particular, diminished recognition of negative facial emotions has been consistently observed in PD patients [3,4,5,6,7,8]. Furthermore, also emotion recognition of circumscribed facial information, e.g., emotional states portrayed only in the eye region is impaired in PD patients [9]. While implicit emotional processing, i.e., the automatic unconscious emotional processing, is largely preserved in PD, as shown by a persistent sensitivity to emotional stimuli [9,10], explicit (conscious) recognition and discrimination of emotional facial cues seem to be generally damaged [1,2,3,4,5,6,7,8].

Deep brain stimulation of the subthalamic nucleus (STN-DBS) is a helpful therapeutic approach to improve motor disturbances in PD [11,12,13] but also to have influences on cognitive and executive domains [14,15,16,17,18]. However, studies investigating the effects of STN-DBS on emotion perception in PD demonstrated rather heterogeneous results, with reports of unchanged explicit emotion recognition of facial expressions (and emotional prosody) under deep brain stimulation (DBS) [18,19,20,21] or worsening of explicit discriminating emotional faces under stimulation [22,23,24]. Precisely, a tendency for DBS to cause deficits in facial discrimination of especially negative emotions as disgust [25,26], anger [27], sadness [28], and fear [26,28,29] was observed. An approach to explain this effect of STN-DBS was given by Geday et al. [23]. The authors stated that stimulation of the STN inhibits the activity in the lateral fusiform gyrus, an area which is generally activated by emotional facial expressions. This would lead to significantly altered emotion perception of facial expressions while emotional assessment per se would not be affected by DBS. Hence, explicit emotional processing would be largely diminished in PD patients under DBS, but implicit emotional processing might not be affected by stimulation.

The studies mentioned above exclusively investigated the influence of STN-DBS on explicit discrimination and rating abilities and left out automatic implicit processing of emotional states. Only Castner et al. [30] measured the outcome of DBS on implicit emotional processing in terms of semantic and affective priming in PD patients. Using verbal material, they found unimpaired automatic (implicit) lexical-semantic and affective processing independent from DBS, while STN-DBS improved controlled, attentional priming in PD patients. The authors suggested that STN-DBS modulates basal ganglia-thalamocortical circuits involved in controlled attentional processes but proposed automatic priming to be unaffected by stimulation. In a previous study [9], we investigated both, implicit and explicit emotional processing of words and circumscribed facial regions in non-stimulated PD patients. Here, results showed that PD patients were impaired in the explicit discrimination of both, emotional words and of emotional facial information that was displayed solely in human eyes. Additionally, largely preserved implicit emotional processing of this stimulus material was found with specific altered processing for the emotions happiness and disgust.

In the present study, we thus assessed the impact of STN-DBS on implicit and explicit processing of emotional words and facial eye cues. We tested patients with an affective priming paradigm and an explicit emotion discrimination task under STN-DBS ON and OFF for the emotions fear, disgust, happiness, and a neutral condition. Based on previous data [9], we expect more pronounced DBS associated alterations selectively for the emotions happiness and disgust.

## 2. Methods

### 2.1. Participants

Fourteen PD patients with DBS of the STN (mean age: 61.9 ± 11.46 years, 4 female; 12 right-handed) were recruited from the Departments of Neurology and Stereotactic Neurosurgery at the University of Magdeburg, Germany. The diagnosis of PD was confirmed by a neurologist specialized in movement disorders. Each patient had been treated with STN-DBS for at least 3 months (mean duration since surgery: 20.86 months (range 3–77 months)). All patients, except for two, were taking supplementary dopaminergic medications in conjunction with DBS (Table 1). All patients were tested under two DBS conditions, while dopaminergic medication was ON (tested conditions: DBS-ON/Med-ON, DBS-OFF/Med-ON). All patients had chosen DBS surgery because their medications were no longer providing optimal control over their motor symptoms.

In all patients, electrodes were placed bilaterally in the STN. The surgical procedure has been described previously [31]. Macroelectrodes (Medtronic Model 3389, Medtronic, Minneapolis, MN, USA) with four platinum–iridium cylindrical surfaces (diameter 1.27 mm, length 1.5 mm, edge-to-edge separation of 0.5 mm) were placed into the STN using MRI-guided stereotaxy and intraoperative microelectrode recordings. The coordinates for macroelectrode placement was based on direct visualization of the STN on T2-weighted magnetic resonance images. Final electrode placement was based on microelectrode recordings and confirmed intraoperatively with macrostimulation after implantation of the DBS electrode. Selection of final bipolar contacts and stimulation settings were based on an individual basis to optimize control over clinically manifested motor symptoms.

Patients were excluded from the study if they had a history of neurological (others than PD), or psychiatric conditions such as untreated or unstable mood disorder, bipolar affective disorder, schizophrenia or other condition known to compromise executive cognitive functioning, or an untreated or unstable medical condition known to interfere with cognitive functioning (e.g., diabetes, pulmonary disease). All patients had normal or corrected-to-normal vision. Prior to participating in the study, all patients provided informed consent. The study was approved by the local University´s ethics committee (number: 96/15, date: 20.08.2015) and was conducted in accordance with the Declaration of Helsinki.

### 2.2. Experimental Procedure

PD patients completed two counterbalanced sessions of two tasks (implicit and explicit task), once with DBS being ON and once with DBS switched OFF. The order of the ON/OFF testing was pseudorandomized across patients (odd-even-even-odd), so that half of the patients started in the ON condition. After switching the stimulator device ON or OFF, respectively, a break of 45 min was included before they started the tasks. Within each DBS session, the order of the tasks was fixed with the implicit task always preceding the explicit task. This ensured that motor symptoms had mainly subsided after activating stimulation and that the increase in motor symptoms had stabilized after stopping stimulation [32,33].

### 2.3. Material

In an implicit lexical decision task (LDT) participants had to evaluate the lexical status of a presented letter string and to decide whether it is a correct word or a pseudo-word. This target word was preceded by an emotionally valenced facial eye region (prime). During this LDT, 192 letter strings (96 German words and 96 pseudo-words) were used. Neutral target words were taken from the “Berlin Affective Word List Reloaded” (BAWL-R) [34] when they showed a valence rating of 0. Emotional target words were extracted from the “Discrete Emotion Norms for Nouns: Berlin Affective Word List” (DENN-BAWL) [35]. Only words were selected with an emotional intensity score equal or superior to 3 according to Briesemeister et al. [35]. The 96 target words consisted of four emotional categories: 24 happiness-related (e.g., “LIEBE”; (“LOVE”)), 24 disgust-related (e.g., “PARASIT”; (“PARASITE”)), 24 fear-related (e.g., “PANIK”; (“PANIC”)), and 24 neutral target words (e.g., “WOCHE”; (“WEEK”)). Pseudo-words were generated from the BAWL-R word list. Here, for neutral words with a length of four to eight letters, single vowels or consonants were interchanged to create new pronounceable but meaningless letter strings (e.g., “POLITIK”—“PILITOK”).

Facial regions around the eyes were extracted from the stimulus set “60 Faces Test” developed by Ekman and Friesen [36] and served as emotional primes. The emotional primes consist of the six basic human emotional facial expressions (happiness, disgust, fear, surprise, sadness, anger and neutral condition). In the present study, we selected four different persons (two female, two male) with their relevant facial expressions (happiness, fear, disgust, neutral). The whole-face images were then adjusted and cut so that only the eye regions were visible. The resulting 16 prime pictures were presented in a size of 8.6 × 3 cm (3.4° visual angle). In the LDT, these eye region pictures (primes) and target words (words or pseudo-words) were combined in a pseudo-randomized manner: half of the prime-target pairs with real German words were constructed to be emotionally congruent, the other half to be incongruent.

### 2.4. Task

The experiment was performed with Presentation (Neurobehavioral Systems, Inc., Albany, CA, USA). Patients were seated in front of a computer and looked at a fixation cross in the middle of the screen.

**Figure 1:**
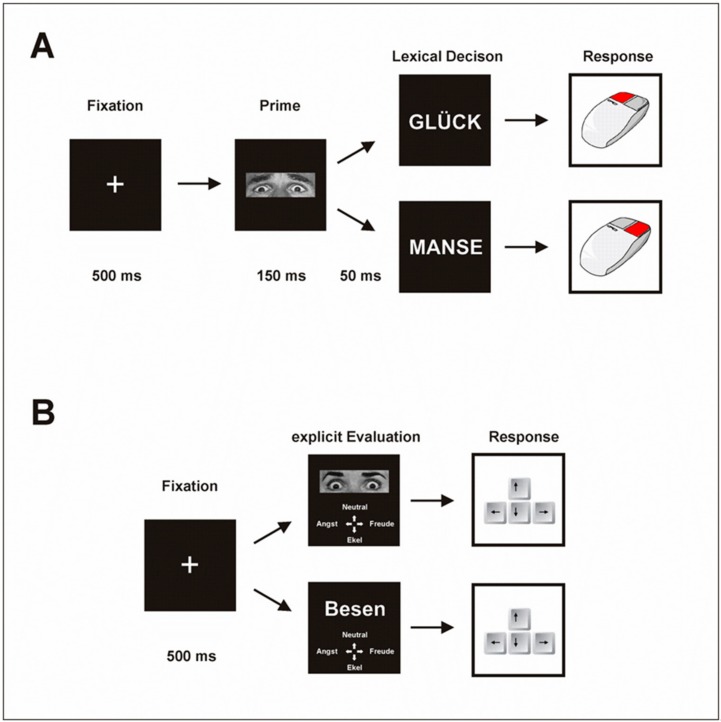
Affective priming paradigm including (**A**) the lexical decision task (LDT) of the implicit task and (**B**) the explicit task with the evaluation of emotional stimuli. (**A**) Each trial in the LDT started with a fixation cross that was presented for 500 ms. An emotional prime (the adjusted eye region of Ekman-faces-photographs) followed and was displayed for 150 ms followed by a break of 50 ms. Subsequently, the target stimulus was presented without a time limit. The participants were supposed to decide as fast as possible whether this target was a real German word or a pseudo-word and to press a corresponding button on a computer mouse. After the button press, the next trial started. (**B**) In the second task, participants were requested to explicitly decide which emotional category the presented stimulus material belonged to. Each trial started with a fixation cross that was presented for 500 ms. After a further 50 ms break, the stimulus was presented. Participants were instructed to press one of the four buttons on a keyboard which corresponded to the emotional category of the stimulus as fast as possible. No time limit was given. First, the eye regions were presented, afterward the real German words followed.

**Implicit task:** The implicit task consisted of 192 trials with a LDT; after half of the trials (96) a break was included; the patients decided how long this break would last. Depending on the understanding of instruction, the length of the break and the response speed of the patients, this task was about 20–25 min. Each trial started with a fixation cross for 500 ms followed by an emotional prime (eye region of a face) displayed for 150 ms. Then the target stimulus (word or pseudo-word) was presented until the patient gave a response. Patients had to decide whether this target word was a real German word or a pseudo-word by pressing the corresponding mouse button. Afterward, the next trial started (Figure 1).

**Explicit task**: The subsequent explicit task consisted of 112 trials and contained the same stimulus material (words and pictures) as in the implicit task (16 faces, 96 real German words). Depending on the understanding of the instruction and the patients´ time to decide, this task lasted about 15 min. The trial started with a fixation cross (500 ms) followed by the presentation of the target (eye or word). First, the emotional eyes were presented, followed by the words. Patients had to explicitly decide which of four emotional categories (fear, disgust, happiness, or neutral) the target stimuli belonged to best. They were instructed to press a corresponding button on a keyboard as fast as possible; however, no time limit was established for the decision. Afterward, the next trial started (Figure 1).

### 2.5. Statistical Analysis

We assessed reaction times (RT) and response accuracies (RA) for all four emotions for button-press answers of patients in the implicit and the explicit task. For the implicit emotional processing, repeated-measures ANOVAs on RT and RA with the within-subjects factors *valence* of the target word (fear/disgust/happiness/neutral), *congruence* between prime and target (congruent/incongruent) and *DBS* (DBS ON/ OFF) were conducted.

For the explicit emotional processing, a repeated-measures ANOVA with the within-subjects factors *valence* of the target word (fear/disgust/happiness/neutral), *condition* of the stimulus (words/faces) and *DBS* (ON/OFF) was applied.

## 3. Results

### 3.1. Implicit Emotional Processing

**Figure 2:**
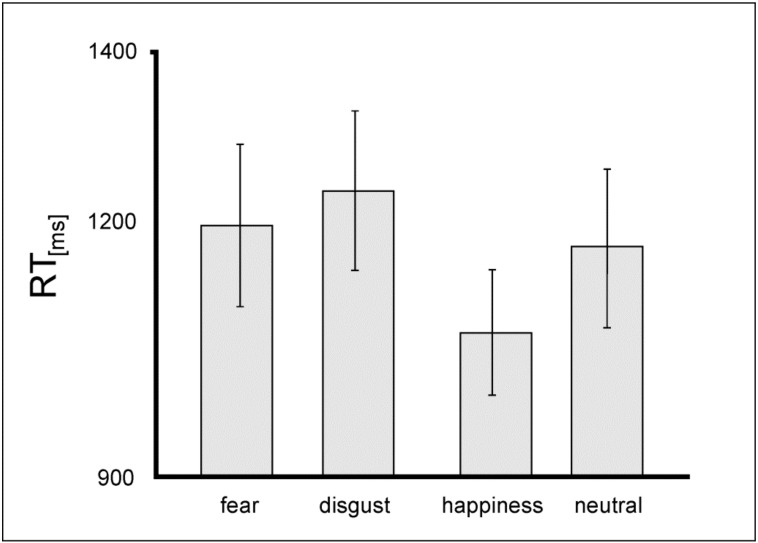
Mean reaction time (RT) values (in ms) for performance in the implicit task for patients. Error bars show standard errors (SE).

**RT**: A repeated-measures ANOVA on RT showed a significant main effect of the factor *valence* [F(3,39) = 7.61; *p* < 0.001] which was driven by longest RT for the emotional category disgust [M = 1233.98 ± 356.49 ms] and shortest RT for happiness [M = 1066.76 ± 283.62 ms], (Figure 2).

**Figure 3:**
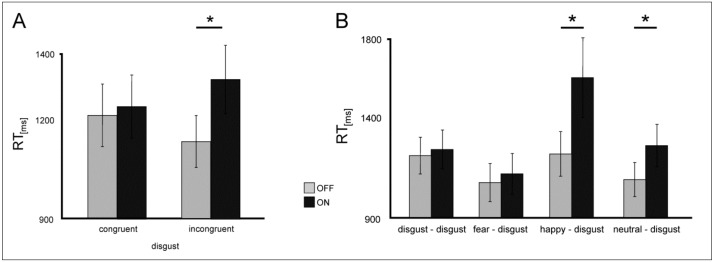
(**A**) Mean RT values (in ms) for congruent and incongruent disgust-connoted stimuli in the implicit task for Parkinson’s disease (PD) patients under deep brain stimulation (DBS) (light grey) and when DBS is switched OFF (dark grey). (**B**) Mean RT values for disgust-connoted prime-target pairs in the implicit task for patients under DBS (light grey) and when DBS is switched OFF (dark grey). Error bars show SE. Significant differences are labeled with (*).

Following our hypothesis, subsequent ANOVAs with the factors *congruence* between prime and target (congruent/incongruent) and *DBS* (DBS ON/ OFF) were conducted for disgust and happiness separately. Analysis revealed no interactions for happiness, but a significant trend for an interaction of the factors *congruence x DBS* for disgust [F(1,13) = 4.19; *p* = 0.06] (Figure 3a). Patients answered incongruently primed disgust-connoted words significantly slower under DBS of the STN (ON vs. OFF: T(13) = 2.3; *p* = 0.039), (Figure 3A). Detailed analysis which was conducted analogously to previous investigations [9] showed that DBS particularly slowed down RT for happy-disgust pairings (ON vs. OFF: T(13) = 2.06; *p* = 0.06) and neutral-disgust pairings (T(13) = 3.05; *p* = 0.009) (Figure 3B).

**RA**: A repeated-measures ANOVA on RA revealed a significant main effect of the factor *valence* [F(3,39) = 6.44; *p* = 0.001] only, which was driven by significantly more errors for disgust. RA rate for disgust was lowest compared to all other emotional categories [M = 79.46 ± 20.2%]. Subsequent separate ANOVAs for disgust and happiness revealed neither significant main effects nor interactions.

### 3.2. Summary Implicit Task

Performance in the implicit task revealed a general valence effect driven by the longest RT and more errors for disgust targets. DBS slowed the processing of incongruently positively or neutrally primed disgust-connoted target words, specifically for positively or neutrally primes.

### 3.3. Explicit Emotional Processing

**Figure 4:**
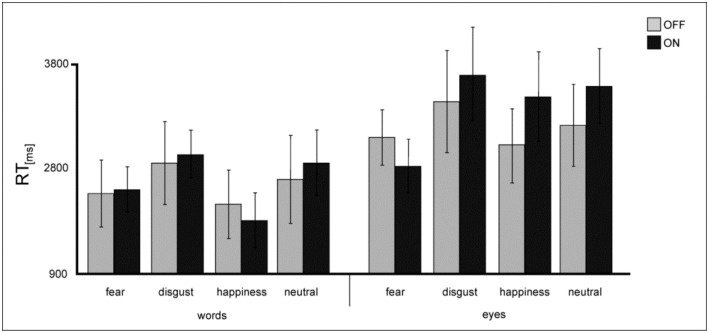
Mean RT values (in ms) of words and eyes for performance in the explicit task for patients under DBS (light grey) and when DBS is switched OFF (dark grey). Error bars show SE.

**RT**: A repeated-measures ANOVA on RT revealed a significant main effect of the factor *valence* [F(3,39) = 6.24; *p* = 0.001] with significantly longer RT for disgust-related stimulus material and a significant main effect of the factor *condition* [F(1,13) = 14.23; *p* = 0.002] showing that answers for words were generally faster than for eyes (Figure 4).

Following our a priori hypothesis, subsequent ANOVAs with the factors *congruence* between prime and target (congruent/ incongruent) and *DBS* (DBS ON/ OFF) were conducted for disgust and happiness separately. Analysis confirmed the main effect for *condition* (disgust [F(1,13) = 10.2; *p* = 0.007], happiness [F(1,13) = 7.25; *p* = 0.018]), and further revealed a significant *condition x DBS* interaction for happy stimuli [F(1,13) = 5.97; *p* = 0.03]. Thus, while for all emotions words were answered faster than eyes, DBS facilitated happy words evaluation and interfered with the processing of happy eyes.

**Figure 5:**
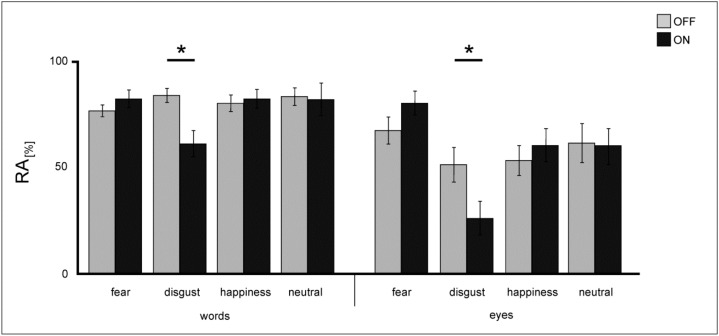
Mean RA values (in %) of words and eyes for performance in the explicit task for patients under DBS (light grey) and when DBS is switched OFF (dark grey). Error bars show SE. Significant differences are labeled with (*).

**Figure 6:**
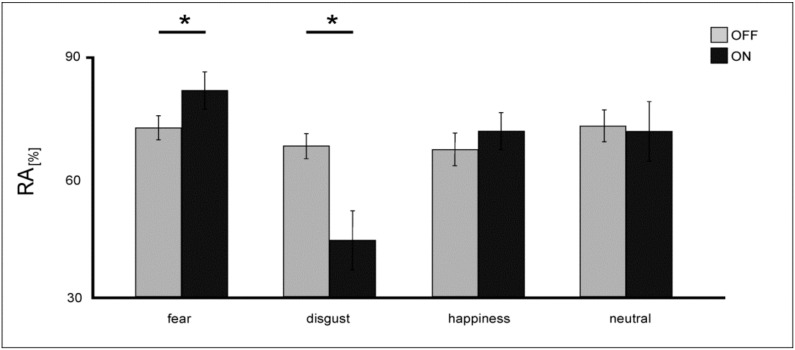
Mean RA values (in %) for explicit evaluation of emotional stimuli for patients under DBS (light grey) and when DBS is switched OFF (dark grey). Error bars show SE. Significant differences are labeled with (*).

**RA**: A repeated-measures ANOVA on RA revealed a significant main effect of the factor *valence* [F(3,39) = 8.48; *p* < 0.001] due to decreased RA rates for the emotional category disgust and a significant main effect of the factor *condition* [F(1,13) = 25.91; *p* < 0.001] showing that eyes generally elicited more errors than words. Analysis also revealed a significant main effect of the factor *DBS* [F(1,13) = 4.84; *p* = 0.04] demonstrating a general decrease in response accuracies under STN-DBS [ON: M = 67.11 ± 17.29; OFF: M = 70.05 ± 15.22]. Furthermore, results showed a significant *valence x condition* interaction [F(3,39) = 4.01; *p* = 0.014], indicating higher RA rates for words than for eyes (Figure 5). Importantly, analysis also revealed a significant *valence x DBS* interaction [F(3,39) = 6.95; *p* = 0.001]: independent from the stimulus condition, DBS of the STN differentially influenced RA rates for selected emotions (Figure 6). STN-DBS ON compared to OFF significantly worsened RA rates for disgust-connoted stimulus material (T(13) = 4.82; *p* < 0.001) and, in contrast, improved RA rates for fear stimuli (T(13) = 2.44; *p* = 0.03).

### 3.4. Summary Explicit Task

Irrespective of DBS mode, patients showed prolonged RTs and highest error rates for the discrimination of disgust stimulus material (main effect of valence). Furthermore, words were generally answered faster than eyes, and eyes elicited more rating errors than words (main effect of condition). Finally, DBS decreased RA rates for emotional ratings (main effect of DBS). Importantly, DBS selectively diminished explicit processing of the emotion disgust but had an ameliorating effect on discriminating fear stimuli (valence x DBS interaction).

## 4. Discussion

In this study, STN-DBS influenced implicit and explicit emotion processing of primed semantic and facial stimuli in PD patients. Depending on the valence of the emotional stimuli and the task condition, patients benefitted or suffered loss from STN stimulation. We observed a general deterioration for disgust-connoted material under STN-DBS but also stimuli-dependent improvement in the explicit processing of fear stimulus material. Thus, STN-DBS did not generally manipulate emotion processing but did selectively interpose according to the valence of the stimuli. Moreover, the observed performance differences under DBS ON when compared to OFF rather apply to RA, although considerable effects of DBS on RT were visible. STN-DBS had no global influence on the speed of responding for all emotions because patients did not generally answer faster or slower under stimulation. Instead, DBS selectively slowed processing of disgust stimuli.

In addition, we found considerably poorer performance in the perception of disgust for both, implicit and explicit emotion processing, and this effect even worsened under DBS. Generally, this finding is in good agreement with recent studies reporting diminished disgust processing under DBS using pictures of whole faces or other stimulus modalities [25,26,37]. We can broaden the state of literature by investigating the influence of STN-DBS on emotion recognition out of eyes only. Aiello et al. [25] found diminished disgust discrimination abilities under DBS for facial expressions and emotional prosodic stimuli in PD patients but emphasized that impaired disgust recognition was prominent also before DBS implant in patients. Impaired processing of disgust thus is related to the neurodegenerative disease itself rather than just to the impact of STN-DBS. Stimulation is not capable to reduce or even eliminate this disgust-related deficit, even when these disgust-associated stimuli are primed with valence-congruent or -incongruent cues. In the present study, we found an inverse priming effect for incongruently primed disgust target words when stimulation was OFF (implicit task). This effect is displayed in terms of shorter RT for these incongruent trials. There are different explanations for the processing advantage of incongruently primed stimuli, see Wagenbreth et al. [38] for a detailed review. DBS neutralized this inverse priming effect by slowing RT for disgust target words, which again emphasizes the deteriorating effect DBS has on disgust perception.

Disgust is an emotion that is evidently hard to process in PD patients, irrespective of DBS mode and even independent of the stimulus extent. Several studies support the special role of disgust [4,10,39,40], indicating it as a negative but nonetheless non-threatening emotion in social contexts. Moreover, the emotion disgust is predominantly affected by the culture a society lives with and is dependent on what people perceive as “disgusting”. A possible contributing and discriminatory factor to our experimental set-up (in which we only use eyes as facial stimuli) might be that disgust —as well as happiness—is mainly and better processed by seeing the mouth of a face, rather than the eyes [41,42].

This might also be a contributing factor to explain our a priori expected but missing significant effects of DBS on happiness processing. We did not find an influence of stimulation on implicit processing and only marginal interfering of STN stimulation on the explicit evaluation of happy words and eyes. Generally, a valence effect for happy stimuli is known, with shorter RT and better RA for positive associated (happiness-connoted) stimuli [35,43]. In accordance, Ibarretxe-Bilbao et al. [44] found a ceiling effect for the recognition of happiness stimuli both for PD patients and for healthy participants. Hence, the processing of happy stimuli is per se easier when compared to negative stimuli (e.g., disgust) and patients might have generally profited from this effect, irrespective of DBS mode. Moreover, Polosan et al. [45] demonstrated that STN stimulation increased positive and decreased negative ratings to low-intensity positive and negative stimuli, suggesting an influence of DBS of the associative-limbic STN on negative cognitive biases and negative appraisals of emotional stimuli.

Functional neuroimaging studies investigating the neural basis of emotion recognition showed the involvement of the amygdala in the processing of fear [39,46,47]. Patients with bilateral amygdala damage, for example, show relatively inaccurate recognizing abilities of fear from faces [48], potentially based on a lack of attention payed to the eye regions of faces. A functional network including the amygdala and both, the orbitofrontal cortex (OFC) and anterior cingular cortex has been suggested to underlie such processing of fearful expressions, with activation of the inferior prefrontal cortex [49,50]. Due to basal ganglia-thalamocortical circuitries, we assume STN-DBS to have an influence on the modulation of the amygdala and suggest a relationship between amygdala functioning and altered fear emotion processing observed in PD patients with STN-DBS. Accordingly, we observed a significant trend for RA improvement of fear words and eyes in the explicit task under stimulation. This is in contrast to previous studies reporting reduced fear recognition in stimulated PD patients [22,29,51] and to other reports showing no effect of STN-DBS on fear processing [19,20].

In this context and as a possible explanation, modifications to the non-motor basal ganglia-thalamocortical circuitry and the emotional functions of the OFC and amygdala through DBS and L-Dopa medication have been proposed. The interaction between L-Dopa and STN-DBS plays a crucial role for patients because in most cases dopaminergic medication intake is continued despite DBS implant in patients. Actually, in the presented PD patient sample, only two out of fourteen patients did not receive supplementary dopaminergic medication to STN-DBS (Table 1). L-Dopa could overdose the mesolimbic projections towards the amygdala and OFC and thus lead to altered amygdala activation in response to emotion perception, for instance of fear [25,37,52]. DBS would compensate for this over-activation by decreasing OFC activity and thereby restoring the necessary OFC-amygdala interaction [26]. L-Dopa would compensate for the decreasing DBS-effect through its respective effects on the OFC and amygdala which may explain the fear recognition improvement when both therapeutic measures are “ON”.

Our results reveal a greater impact of DBS on explicit rather than on implicit processing. Investigations of DBS outcome on implicit emotion processing are rare. To our knowledge, only Castner et al. [30] measured the influence of DBS on implicit emotional processing in terms of semantic and affective priming in PD patients but used words as stimulus material, not faces. The authors reported preserved automatic lexical-semantic and emotional processing in PD patients with STN-DBS, but no direct influence of DBS on implicit processing was detected, which is mostly likewise to our results. Due to preserved priming effects for short stimulus onset asynchronies in PD patients, irrespective of STN-DBS conditions, Castner and colleagues concluded that the automatic activation of emotional evaluations is unimpaired in PD, which would be due to the functioning of basal ganglia-thalamocortical circuitry, linking structures important for facial emotion recognition. The disruption of these loops through neurodegenerative processes is thought to contribute to the accompanying cognitive decline in PD, including impaired emotional processing abilities [53]. Because implicit verbal processing in PD is intact even under STN-DBS, it can be concluded that the basal ganglia-thalamocortical circuits are likely not to be involved in the automatic activation of emotion evaluations. We can expand these findings for nonverbal circumscribed facial stimulus material. Moreover, we assume that implicit emotional processing is still intact in PD since it requires no cognitive demands as it is the case in conscious explicit discrimination of emotions. Basal ganglia-thalamocortical activation would be necessary if cognitive-driven decisions are requested. Since the effect of STN-DBS was more pronounced on explicit rather than on implicit emotion processing in the present study, we can confirm this assumption.

Methodological differences (e.g., medication states, early vs. late PD stages of the patient samples, different times since surgery) might have contributed to the observed differences between our and recent studies. However, we would like to stress the selective influence STN-DBS can have on emotion discrimination and the importance to differentiate between implicit and explicit emotion processing. Future studies investigating the impact of DBS on emotion discrimination out of single facial regions might help to shed light on the efficacy of STN stimulation in emotion perception. The findings of this present study contribute to the importance of investigating DBS outcome in patients in the context of clinical care and post-surgical support. We demonstrated deteriorated recognition of disgust out of faces and words after DBS surgery. This might have implications for communication and social living for relatives, nurses or caregivers of patients. Precautionary arrangements and accords should be entered to ensure optimal cooperation and well-being for patients and to avoid misconceptions when dealing with disgust-connoted objects. Patients should be supplied with adequate information about the possible side or non-motor adverse effects after STN-DBS, like possibly deteriorated processing abilities of negative emotions, which, for example, might lead to an incautious handling of disgust-associated and possibly dangerous materials.

The reported findings have to be regarded under the following possibly constraining aspects. First, we tested patients under supplementary medication which might have influenced the results [54]. However, measures with and without DBS were applied under the same medications states, so results for both conditions should be comparable. Second, pre-surgical testing of emotion recognition would have been favorable to directly draw comparisons over time and surgery. Our study provides information of a rather small sample size with fewer women than men. In future studies, a gender- and age-matched patient sample would be recommended to further assess differences in emotion performance after DBS.

## 5. Conclusions

In summary, this is the first study to investigate the influences of STN-DBS on implicit and explicit emotion processing in patients with PD. DBS affected explicit emotional processing more than implicit processing and had a more pronounced effect on RA than on RT. While STN stimulation generally impeded the processing of disgust-connoted stimuli, we found an ameliorating effect on RA rate in the explicit evaluation of fear stimuli.

## Figures and Tables

**Table 1:** Demographic and clinical characteristics of patients.

Patient #	Gender	Age	Disease Duration (years)	Time Since Surgery (months)	LED (mg)	UPDRS- III ON	UPDRS- III OFF	DBS Contacts (l/r)	DBS Voltage (V), Frequency (Hz), Pulse Width (μs) left/right
**1**	female	74	16	3	225	16	38	1 − G+ / 10 − G+	2.5 V, 130 Hz, 60 µs / 2,0 V; 130 Hz, 60 µs
**2**	female	59	13	4	500		20	2 − 3 − G+ / 10 − 9+	2.8 V, 60 Hz, 210 µs / 1,5 V, 60 Hz, 210 µs
**3**	male	67	18	77	630	9		3 − G+ / 4 − 5 − 6+	4.8 V, 130 Hz, 60 µs / 3,4 V, 130 Hz, 60 µs
**4**	male	65	16	15	325	18	32	G+ 0 − / 8–9 − G+	2.7 V, 150 Hz, 60 µs / 3,1 V; 150 Hz, 60 µs
**5**	female	71	20	7	350	23	38	1–2 − G+ / 10 − G+	1.3 V, 130 Hz, 60 µs / 1,5 V, 130 Hz, 60 µs
**6**	male	61	10	16	850	12	32	2 − G+ / 10 − G+	3.6 V, 110 Hz, 90 µs / 3,4 V, 110 Hz, 90 µs
**7**	female	66	11	77	200	16	27	1 − G+ / 6 − G+	2.0 V, 190 Hz, 60 µs / 2,5 V, 190 Hz, 60 µs
**8**	male	63	10	4	200	4	19	2 − G+ / 10 − G+	3.1 V, 130 Hz, 90 µs / 3,1 V, 130 Hz, 90 µs
**9**	male	36	6	14	0	13	38	2 − G+ / 10 − G+	2.5 V, 200 Hz, 90 µs / 2,7 V, 200 Hz, 90 µs
**10**	male	53	10	54	385	15	29	1 − 2+ / 9 − 11+	2.5 V, 180 Hz, 60 µs / 4,4 V, 180 Hz, 60 µs
**11**	male	74	13	8	800	22		1 − G+ / 5–6 − G+	4.5 V, 130 Hz, 60 µs / 5,0 V, 130 Hz, 60 µs
**12**	male	41	7	6	350	19	32	2 − G+ / 8–10 − G+	2.0 V, 130 Hz, 60 µs / 2,1 V, 130 Hz, 60 µs
**13**	male	66	8	3	600	11	26	0 − G+ / 8 − G+	1.5 V, 130 Hz, 60 µs / 1,5 V, 130 Hz, 60 µs
**14**	male	70	6	4	0	17	24	1 − 2 + / 9 − 10 +	3.5 V, 130 Hz, 60 µs / 3,5 V, 130 Hz, 60 µs

LED = L-Dopa equivalent daily dose in mg. UPDRS-III = Unified Parkinson disease rating scale − III (motor evaluation).

## Abbreviations

ANOVA	Analysis of variance
BAWL-R	Berlin Affective Word List Reloaded
DBS	Deep brain stimulation
LDT	lexical decision task
MRI	Magnetic resonance imaging
OFC	orbitofrontal cortex
PD	Parkinson’s disease
RA	response accuracy
RT	reaction times
STN	Subthalamic nucleus
